# The Contribution of Trees and Green Spaces to Household Food Security in eThekwini Metro, KwaZulu-Natal

**DOI:** 10.3390/su15064855

**Published:** 2023-03-09

**Authors:** Qhelile Ntombikayise Bhebhe, Mjabuliseni S. C. Ngidi, Muthulisi Siwela, Temitope O. Ojo, Simphiwe Innocentia Hlatshwayo, Tafadzwanashe Mabhaudhi

**Affiliations:** 1 African Centre for Food Security, School of Agricultural, Earth and Environmental Sciences, College of Agriculture, Engineering and Science, University of KwaZulu-Natal, Private Bag X01, Scottsville, Pietermaritzburg 3201, South Africa; 2 Centre for Transformative Agricultural and Food Systems, School of Agricultural, Earth and Environmental Sciences, College of Agriculture, Engineering and Science, University of KwaZulu-Natal, Private Bag X01, Scottsville, Pietermaritzburg 3201, South Africa; 3 Department of Agricultural Extension and Rural Resource Management, School of Agricultural, Earth and Environmental Sciences, College of Agriculture, Engineering and Science, University of KwaZulu-Natal, Private Bag X01, Scottsville, Pietermaritzburg 3201, South Africa; 4 Dietetics and Human Nutrition, School of Agricultural, Earth and Environmental Sciences, University of KwaZulu-Natal, Private Bag X01, Scottsville, Pietermaritzburg 3201, South Africa; 5 Department of Agricultural Economics, Obafemi Awolowo University, Ile-Ife 220101, Nigeria; 6 Disaster Management Training and Education Centre for Africa, University of the Free State, Bloemfontein 9301, South Africa; 7 International Water Management Institute (IWMI-SA), Southern Africa Office, Pretoria 0184, South Africa

**Keywords:** HFIAS, food security, trees and green spaces, households, Instrumental Variable Poisson model

## Abstract

One of the most significant issues faced by many low- and middle-income nations, including South Africa, is ensuring access to healthy, affordable, and sustainable food. South Africa is renowned worldwide for its rich biodiversity and a vast body of traditional knowledge among those who consume forest foods. However, despite ecological diversity, frequent barriers remain to getting diversified household diets. This study sought to investigate the contribution of trees and green spaces to household food security in eThekwini. A total of 280 households met the inclusion criteria and consented to participate in this study by responding to questionnaires. The collected data were analysed using descriptive statistics, the computation of the Household Food Insecurity Access Scale (HFIAS), and the Instrumental Variable Poisson model. The study’s results revealed that only 29% of the respondents were food secure, 36% were mildly food insecure, 27% were moderately food insecure, and 8% were severely food insecure. The Instrumental Variable Poisson model results revealed that cultivated green spaces, wealth index, gender, education level of the head of households, and grants had a negative correlation with household food insecurity. On the other hand, non-cultivated green spaces, local trees, age, marital status, number of dependents, and monthly income positively correlated with food insecurity. Given the existence of trees and green spaces in eThekwini, there is potential for food security solutions to be formed around both cultivated and uncultivated green spaces to promote sustainable access to food and nutritious diets in low-income households. Policy interventions should adopt an approach that encourages the incorporation of foods from both cultivated and uncultivated trees and green spaces in people’s diets.

## 1 Introduction

The South African food system faces wicked challenges; the country has the highest rates of wealth inequality in the world, and there are growing concerns about poverty [1,2]. It is difficult to transition to a healthier, more sustainable, and equitable food system in these circumstances. As a result, despite global efforts to end poverty and hunger, South Africa continues to face numerous socio-economic challenges, such as unaffordable healthy diets for many poor households [3–5]. More than 2.3 billion people worldwide were food insecure as of 2021, indicating a rise of 15% from 2019 [6]. Presently, more than half a billion people in Africa do not have access to food that guarantees an active and healthy life [7]. More than a quarter of African children are stunted, and about a third experience micronutrient deficiency. Unacceptably high levels of poverty, unemployment, and inequality are prevalent in South Africa. A sizable section of the population suffers from food insecurity and non-communicable diseases caused by unhealthy diets [6,8].

EThekwini is situated in KwaZulu-Natal, one of South Africa’s nine provinces with a population of over 11.1 million people. Over a third of South Africa’s child hunger is in KwaZulu-Natal, two to four times more prevalent than in any other province [7,9,10]. Food insecurity, hunger and malnutrition, a lack of nutritional variety, and increased disease vulnerability are all effects of a failed food system [7]. Numerous intricate and interconnected causes, including climate change, insufficient health care, population increase, social and political dynamics, rapid urbanisation, and economic inequality, contribute to the ongoing hunger, food insecurity, and malnourishment [11,12].

Very undesirable demographic, economic, cultural, and environmental changes are currently occurring globally. These changes have had catastrophic impacts on human communities and the ecosystems on which they rely for food and livelihoods [12]. Ecosystem degradation and increase in poverty and food insecurity levels are among the most significant undesirable challenges in Africa. There has been a significant disruption of food systems, further exacerbating the already stated socio-economic crises. These crises have stalled the efforts to meet the Sustainable Development Goals (SDGs) and have had dire effects on food and nutrition security [12–15]. Despite the introduction of critical global action frameworks, particularly the Sustainable Development Goals (SDGs), the world is still struggling to balance food needs with the Earth’s available natural resources. The use of trees and green spaces to alleviate food insecurity could contribute to the achievement of SDG 2.

Food and nutrition security, achieved when all members of a household consume adequate food to meet their individual dietary needs [11], is one South Africa’s priorities [12]. There is an increased expectation for population growth and global demand for food in the coming decades. On the other hand, uncertainty surrounds the capacity of all trees and green spaces to help sustainably supply food demands [13,14]. It is possible to eradicate hunger by paying greater attention to what trees and green spaces can contribute towards a food-secure South Africa. Trees and green spaces can contribute towards ensuring the attainment of the four pillars of food security (access, availability, utilisation, and stability) while facilitating the consumption of nutritionally adequate diets in quantity, variety, diversity, and nutrient content [2]. More interventions are needed in acknowledging the role trees and green spaces can play in achieving food security, reducing poverty, and improving livelihoods.

Trees and green spaces are any open and vegetated green areas that have the potential to contribute to the overall quality and sustainability of household diets [15]. These spaces include forests, parks, grasslands, croplands, wetlands, savannahs, and other terrestrial spaces covered with vegetation and trees [16,17]. They can be in the form of (i) productive green spaces (farms, gardens, nurseries), (ii) recreational green spaces (parks and play areas, (iii) natural and semi-natural green spaces (wetlands, woodlands, grasslands and bushlands), and (iv) linear green spaces (all kinds of vegetation along roads and along riverbanks) [14,15].

The importance of trees and green spaces and their contribution to food security is increasingly recognised worldwide [2]. This is due to the fact that trees and green spaces, although often undervalued, play a key role in supporting food and nutrition security in all. They provide (i) nutritious and diverse foods (which include, nuts, leaves, tubers, insects, and mushrooms) and feed for livestock; (ii) energy for cooking; (iii) both formal and informal employment; and (iv) ecosystem services necessary for agriculture and food production now and in future. The contributions of trees and green spaces to food and nutrition security should be considered vital and incorporated in the policies that are aimed at achieving Sustainable Development Goals 2 and 15, both of which are linked to food and nutrition security as well as trees and green spaces [18–20].

**Figure 1:**
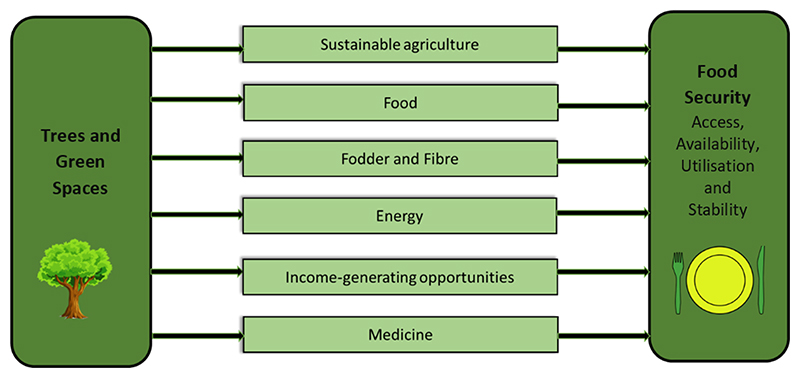
Multiple contributions of trees and green spaces to food security and nutrition (Source: Author).

Therefore, there is a need to develop a food system that is good for the people and the ecosystem by formulating holistic solutions to solve the food insecurity issue. One such move is to advance sustainable food systems by including all vegetation—cultivated and uncultivated—in people’s diets to reduce the pressure on farming to solve food insecurity. Figure 1 illustrates how trees and green spaces, directly and indirectly, contribute to food security.

Africa’s population is predominantly rural and relies strongly on trees and green spaces for their livelihoods. However, trees and green spaces’ role in alleviating poverty and contributing to food security is still under scrutiny and has not been explicitly explored [20]. Scholars have dismissed increasing crop production through conventional means as a critical solution to food security [18,21]. Achieving food security goes beyond placing control in the hands of the farmers [22–24]. Most studies [23,25] have focused more on the impact of staple crops on food security; hence, there is limited empirical research on the actual contribution of all trees and green spaces to food security. Incorporating cultivated and uncultivated green spaces into the food system is still emerging, globally. However, in the recent past years, many research publications and two major global reports have drawn attention to the contributions of trees and green spaces to food and nutrition security [26]. This is leading to a better recognition of these roles by international organisations, such as the United Nations Committee on World Food Security (CFS) and the Food and Agriculture Organisation (FAO).

Therefore, this research seeks to contribute to this growing knowledge base on how cultivating and uncultivated green spaces can lead to sustainable food security.

This study aims to assess the impact of local trees and green spaces on household food security. It hypothesises that trees, green spaces, and food and nutrition security are linked. Such linkages help to plan interventions that could improve food security in low-income and poverty-stricken communities.

The current study report is structured into five sections. The introductory section is followed the methodology section, which includes a description of the study area, data collection methods, and the conceptual framework. The third section presents the research findings, focusing on the contribution of trees and green spaces to household food security. The fourth section discusses the findings of interviews, focus groups, and empirical frameworks. The conclusion and recommendations are presented in the last section.

## 2 Methodology

### 2.1 The Study Setting

**Figure 2:**
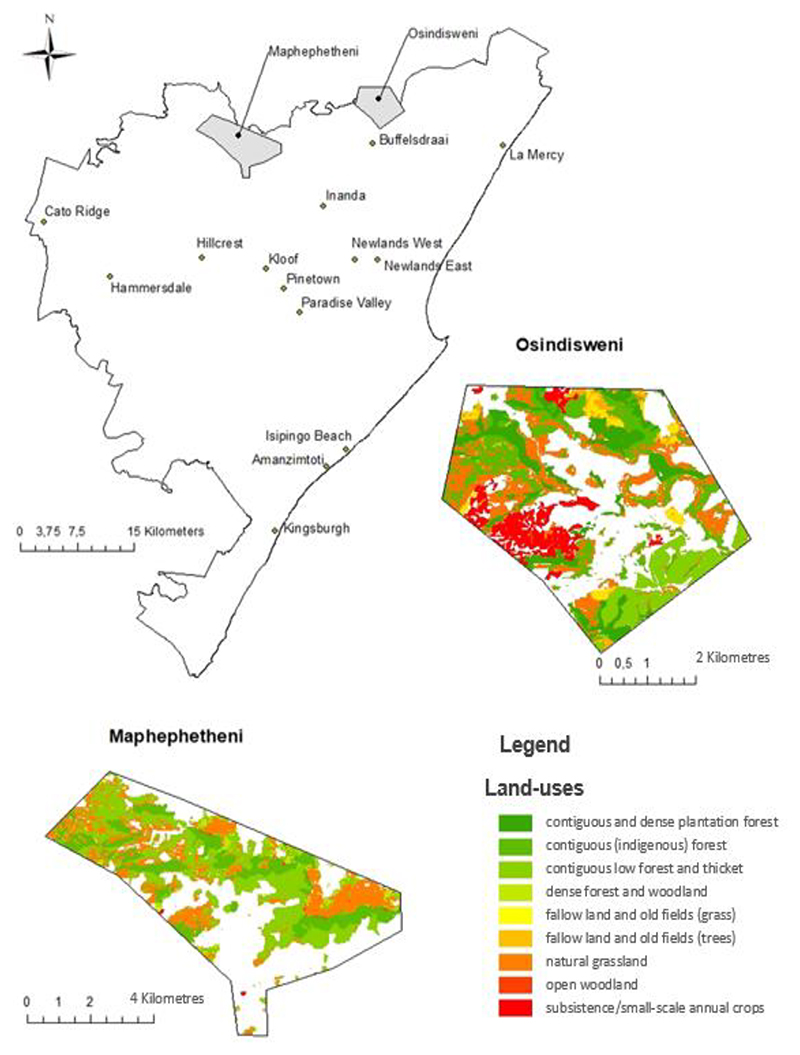
Map showing the distribution of green spaces within the Osindisweni and Maphephetheni study sites in KwaZulu-Natal, South Africa (Source: Author).

This study analyses how trees and green areas contribute to food and nutrition security in communities within the eThekwini Municipal, a portion of the province of KwaZulu Natal [26]. The EThekwini Municipal Area (EMA) encompasses the communities of Osindis-weni and Maphephetheni, where data were collected. The two study sites were specifically chosen because they are similar and are thought to be biologically varied locations with a lot of vegetation (forests, grasslands, woods, and bushlands) [27,28]. Africans mostly populate the communities and are associated with severe poverty, high unemployment, ecological damage, and food and nutrition insecurity [29]. This has demonstrated the necessity of several socio-economic programs, which use research to try and eradicate poverty and restore ecosystems while also assuring food security [30,31]. The distribution of green areas at the KwaZulu-Natal, South Africa, study sites of Osindisweni and Maphephetheni is depicted in Figure 2 below.

### 2.2 The Selection of Respondents

For the initial sampling phase, Osindisweni and Maphephetheni Uplands were specifically chosen owing to their levels of poverty, food insecurity, ecosystem projects, and opportunities available to them. A total of 244 households were included in the sample after it was calculated using the Raosoft sample size computation from 2004 (90% confidence level).

Stratified sampling was then done where the population was split into two strata, each represented by a separate area, Osindisweni and Maphephetheni. As a result, the sample size for each study area was calculated depending on its proportion to the overall sample size.

Sample sizes for Osindisweni and Maphephetheni were 41 and 203, respectively, and the sample size derived was a total of 244 households. However, the enumerators managed to collect data from 280 households, due to the willingness of the respondents to participate in the study. The last and final sampling stage was random sampling, each study area had households, and the respondents were selected randomly on the ground.

### 2.3 Research Design

This paper’s research data were obtained between June and December 2021. Standardised household questionnaires were used to collect data. The questionnaire included questions regarding specific subjects, such as demographic and employment information. It had open-ended and closed-ended questions to gauge respondents’ attitudes and opinions regarding the value of trees, green spaces, and food and nutrition security. Face-to-face interviews were conducted during the surveys—one individual from each household was interviewed by a trained enumerator. All ethical requirements were followed before, during, and after the data collection. Three multilingual (isiZulu and English) enumerators administered the questionnaires.

### 2.4 Data Analysis

The Statistical Package for Social Sciences (SPSS) version 27 analysis was used to manage (capture, code, and clean) the data after they had been collected. The socio-economic makeup of the respondents and the research areas was described using descriptive analysis. To determine whether there were significant correlations between trees and green spaces, household food security, and nutrition, the Poisson regression analysis was then done. The HFIAS indices were calculated to measure the communities’ household food security status.

#### 2.4.1 Computing the HFIAS

The Household Food Insecurity Access Scale, a globally recognised food measurement tool, was used in the food security assessment (HFIAS). The HFIAS was used to assess the “access component of household food insecurity” using data from the previous four weeks (month). The HFIAS instrument was developed by Coates et al. [32] and this scale contains about nine questions about household food access, and the responses are usually provided by the household head [32,33]. The HFIAS score indicates the extent to which a household experienced food security or insecurity four weeks before the study was conducted. The computation of an HFIAS score involved combining the codes for each item that dealt with the frequency of occurrence for that particular household. The HFIAS score is between 0 and 27. A household with a score higher than the national average may be facing food insecurity. A household with a lower-than-average score may also be considered food secure [32]. The HFIAS score is determined using the equation shown below:

$$\text{Average}\;\text{HFIAS}\;\text{score}\;=\frac{\;\text{Sum}\;\text{of}\;\text{HF}\;\text{IAS}\;\text{in}\;\text{the}\;\text{sample}}{\;\text{Total}\;\text{number}\;\text{of}\;\text{HFIAS}\;\text{scores}\;\text{in}\;\text{the}\;\text{sample}}$$

#### 2.4.2 The HFIAS Survey Questions

The participants were asked the HFIAS’s nine questions. The aim of the survey was to determine whether participants had experienced any difficulties in obtaining food in the previous four weeks. The questions were divided into three sections that demonstrated an increasing level of severity of food insecurity (Question 1), inadequate food quality (Questions 2–4), and insufficient food intake (Questions 5–9). The participants were asked to specify how frequently the situation occurred, i.e., if it occurred rarely (once or twice) or never, occasionally (three to ten times), or frequently (more than ten times in the past month). Table 1 below shows the frequencies and percentages of food insecurity situations.

#### 2.4.3 The Instrumental Variable Poisson Model

The HFIAS was used to assess food security. Following that, an Instrumental Variable Poisson model was chosen because it appropriately captures the dependent variable’s count nature. The model is widely used to model non-negative outcome variables and to represent count outcomes. The instrumental variable model estimates the parameters of a Poisson regression model with endogenous regressors [34], to estimate the model’s parameters, instrumental variables that do not correlate with the error term are used in this case. These instrumental variables are related to the endogenous variables but not the model’s error term. Either additive or multiplicative error terms may be used to specify the model. In the error form representation of the exponential conditional mean model, the dependent variable, *FS*_*i*_, is a function of exogenous covariates, *X*_*i*_, endogenous covariates, *Z*_*i*_, and error term, ∈_*i*_. An instrumental variable can enter either additively or multiplicatively, as shown in Equations (1) and (2), respectively [35].

(1) $$FS_{i}=exp\left(X_{i}\;\beta_{i}+Z_{i}.\omega_{i}\right)+\in_{i}$$

(2) $$FS_{i}=exp\left(X_{i}\;\beta_{i}+Z_{i}.\omega_{i}\right)\in_{i}$$

Following Ndlovu et al. [34], three indices, cultivated trees and green spaces, uncultivated trees and green spaces, and local trees, were developed for analysis. The outcome demonstrates that the indicators’ dependability is suitable for examining how trees and green spaces affect households’ food security. The indices created were then employed as independent variables in an Instrumental Variable Poisson model, among other factors.

## 3 Results

### 3.1 Socio-Demographic Profiles of the Respondents

The findings of the study show that more women than men took part in the study, 81.6% and 73.5% in Osindisweni and Maphephetheni, respectively. As a result, women likely head the majority of homes in Osindisweni and Maphephetheni. Most homesteads’ household composition was nuclear; over 90% of families in the study area were nuclear and less than 6% of the homesteads were polygamous. In Osindisweni, 65.8% of respondents and in Maphephetheni, 57.4% were married. For both communities, most respondents had completed secondary education. Less than 25% of respondents, however, had obtained tertiary education, either in the form of a certificate or a diploma. Fewer than 10% of respondents said they had no formal schooling. This information is displayed in Table 2 below, and the findings support the view that Osindisweni and Maphephetheni have restricted access to chances for formal education, employment, and income-generating opportunities.

Trees and green spaces contribute indirectly to food security through employment and income generation. However, in the areas covered by the current study, tree-related jobs did not represent the main source of livelihoods. This was not expected given that most households in the community were poor and vulnerable. Most respondents in the study area had one source of income, and only 4% of the households employed several cash income sources for their livelihood. As depicted in Table 1 above, cash income from social grants and employment accounted for the largest contributor to household income, followed by income from family, friends, and jobs. The other income contributors were income from crops, fruit, livestock, traditional medicine, and fuelwood sales. More than 70% of the respondents in Osindisweni and 40% in Maphephetheni had at least one individual receiving social grants. Most included child support and old age grants, which amounted to ZAR 270 monthly. Some households had members involved in both full and part-time employment. However, the proportion of employed individuals was very low. The people employed were engaged in low-paying jobs as suggested by the monthly income bracket of the respondents ranging from ZAR 0-15,000. Only 3% of the respondents earned more than ZAR 9000, and most of them are employed as teachers in schools surrounding the communities. The results indicate that 10% of the respondents had no income, which was concerning because all the respondents were heads of households.

### 3.2 Household Food Insecurity Prevalence

**Figure 3:**
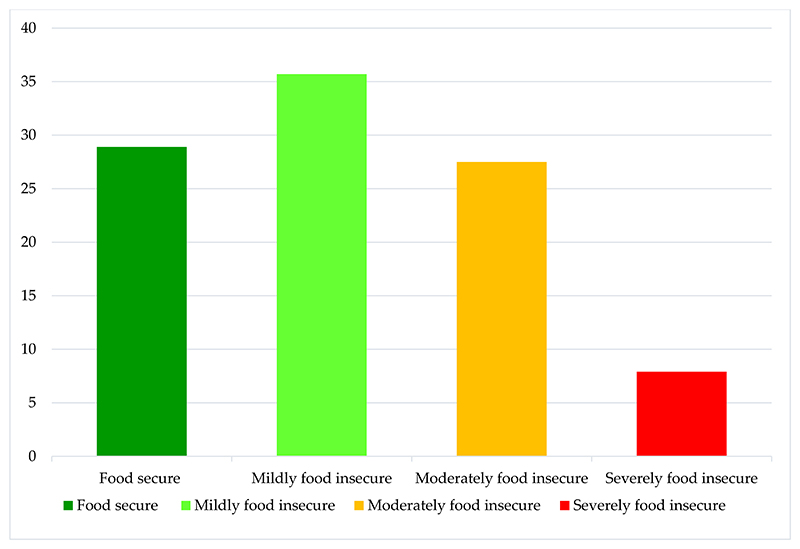
The percentage distributions of the household food insecurity access score (Source: Own analysis).

The HFIAS measuring households’ access to food revealed that only 29% of the respondents were food secure, 36% were mildly food insecure, 27% were moderately food insecure, and 8% were severely food insecure in the study areas, as illustrated in Figure 3 below. These results show that the majority of the households were food insecure.

These results are in line with the results shown in the HFIAS responses in Table 1, 6.4% to 62.5% of the study participants answered “yes”, 11.8% to 31.1% answered “rarely”, 2.1% to 25.7% answered “sometimes”, and 1.8% to 5.7% answered “often”. The affirmative response “yes” had the highest frequency and percentage, which showed mild to moderate forms of food insecurity such as to “worry about not having enough food”, “being unable to eat the kinds of food you prefer”, and “eating smaller of fewer meals per day”. The response “often” had the least frequency and percentage and showed severe forms of food insecurity; it was worrying to note that some households can go a whole day and night without food.

Household food insecurity prevalence.

### 3.3 The Instrumental Variable Poisson Model

According to the Instrumental Variable Poisson model results, eleven variables had a statistically significant influence on household food insecurity, as shown in Table 3 below. Five significant variables were found to be negatively correlated with household food insecurity, implying that increasing either would result in a decrease in food insecurity, suggesting an increase in household food security level. Six of the significant variables, on the other hand, were significant in the positive direction, implying that an increase in any of these variables would be associated with a decrease in food security, suggesting an increase in food insecurity or a less food-secure household.

## 4 Discussion

Food insecurity is an issue that many low- and middle-income nations, including South Africa, face [2]. In this study, more than half of the respondents were food insecure as shown in Figure 2. When asked about the sources of their regular meals, more than half of the respondents stated that they had bought from markets and spaza shops. Furthermore, 12% and 28.8% from Osindisweni and Maphephetheni had harvested from home gardens and green spaces around them, and friends and relatives had assisted less than 25% of the respondents. Out of interest, respondents were asked about their most preferred sources of food, and some respondents agreed that trees and green spaces diversify their diets, and they also confirmed that they preferred food harvested from trees and green spaces instead of store-bought food. Thus, it is evident that trees and green spaces contribute to household diets.

The regression analysis results (Table 3) revealed that cultivated green spaces had a negative and significant impact on household food insecurity. The analysis shows that as the use of cultivated green spaces for food increases by a unit, household food insecurity decreases by 0.097, with all other factors held constant. These results also show that households that harvested food from trees and green spaces had more food access than households that did not utilise green spaces for food. The probable explanation for these results might be that 88.4% of the households had trees in their yards and produced food (vegetables, fruits, tubers (sweet potato and amadumbe—*Colocasia esculenta*), legumes, and maize) in their home gardens and farms, which helped to diversify diets. Some households also indicated that they had livestock in their homesteads which was also the primary source of protein in the form of meat and eggs. In the same vein, the study by Cheteni et al. [36] found that households that grow and cultivate plants for food improved their food security status because of increased production, income, and consumption. Likewise, in South Africa, based on the various studies conducted in different regions of the country, cultivating plants for food through gardening and farming has increased livestock production, crop diversification, and intensification [36–38]. These outcomes, in turn, contribute to assured food security.

Uncultivated green spaces and local trees had a negative and significant relationship with household access to food. This implies that food access from uncultivated trees and green spaces (forest foods) increased food insecurity. This result was surprising and unexpected because forest foods provide many benefits to households in their vicinity, such as food, medicine, and fuelwood for food preparation. This result might have been because of a lack of scientific knowledge on the available food resources in forest trees and green space, use potential, changes in diet, and their harvesting and preparation techniques.

The coefficient of the age of the household was positive and statistically significant in influencing the food insecurity status of the households. This result implies that as the age of the household head increases by a unit, the household food insecurity increases by 0.006, with other variables in the model held constant. A decrease in the age of the household head results in a reduction in food insecurity. The younger the household head, the lesser the food insecurity; this might be because younger people have access to more income-generating opportunities, which enable them to have funds to access food, especially in markets. However, this finding is contrary to Ndlovu et al. [34] and Poppy et al. [20], who found that household food insecurity decreases as age increases because the older population knows about cultivating their own food and the harvesting and preparation of indigenous foods.

The coefficient of the wealth index for both agricultural and non-agricultural related assets was negative and statistically significant in influencing the food insecurity status of households. This could be attributed to the fact that tools and assets such as the radio and television enhance technology adoption, awareness, and spread of information resulting in increased food production and access to income-generating opportunities which all improve access to food [34,38]. About 20% of the respondents confirmed that they sometimes get information on radio and television that helps them produce more food or access food.

The results in Table 3 also show that the coefficient of income was positive and statistically significant. This implied that the higher the income, the higher the household food insecurity. A positive relationship was not expected because income improves access to and food availability. However, this could be attributed to the increase in the cost of living and food in South Africa; hence, more income will not always lead to improved access to food and nutrition. Ndlovu et al. [39] found a positive relationship between income and food access. On the other hand, Gebreyesus et al. [38] conducted food security studies in the Eastern Cape province in South Africa and found that households where the head of household is employed have a much higher income and are more likely to have acceptable diets and a better food and nutrition status. The results show that, to a lesser extent, there is a reliance on trees and green spaces as well as other goods and services that the ecosystem provides. Less than 2% of the households as shown in Table 1 were generating income through the sale of tree products such as herbs, medicine, and fuelwood. However, the total income from those sales was minimal (less than ZAR 1000 a month). This differs from Angelson’s [40] study findings which revealed that income from tree products is often higher or equivalent to income from agriculture. This suggests that trees and green spaces can sustain household income and reduce pressure on the government to provide social grants [40–42]. The money the government uses for grants can be saved for other development purposes. Hence, emphasis on deriving income from products from trees and green spaces can encourage households to plant more trees on their homesteads and conserve the green spaces surrounding them [36,40]. If this is not applied, the quantity of tree products that households purchase may continue to increase, leading them to higher food insecurity given their low income.

This study established a negative and significant association between social grants and the insecurity status of the household. This implies that receiving grants reduces food insecurity. This result was expected because social grants in South Africa have improved food consumption and the poverty status of most households. However, poor diets continue to be a massive challenge for South Africans, especially those living in rural communities; this has been further exacerbated by the increased cost of living [36]. Contrary to these findings, Cheteni et al.’s [36] research in eThekwini revealed poor diets and nutrition insecurity among households reliant on social grants for income. Most respondents who had access to social grants were food insecure because the money received was too little to cover the household food needs. Similarly, Kamanga [41] conducted a study on rural livelihoods and reported that most households’ main income source was grants. There is a deep dependence on grants, which is explained by the fact that many people in the study are unemployed or earn very low cash income. Withdrawal of social grants would worsen the food insecurity situation of many residents.

Table 3 shows that formal education had a negative and statistically significant influence on household food insecurity. The marginal analysis shows that as a household’s formal education increases by one unit, household food insecurity decreases by 0.072 while all other variables in the model remain constant. The relationship was anticipated because formal education increases human capital and knowledge, increasing access to opportunities and contributing to the availability and accessibility of diverse foods at all times. This finding is consistent with the findings of a study conducted in South Africa’s Limpopo province by De Cock et al. [43], who found that households with educated household heads were less likely to be food insecure, and that promoting formal education in rural households can significantly improve food security levels.

The variable of gender had a negative relationship with a household’s food insecurity. This points out that gender affects access to nutritious food. Compared to single-head households, nuclear and female-headed households had better diets and higher incomes. Kennedy et al. [44] notes that this is the case since women are responsible for food preparation and spend the majority of household income on high-quality, protein-rich foods [44]. Additionally, Taruvinga et al. [45] discovered that households with female heads had healthier diets than those with male heads [45] because they were involved in sourcing and preparing food.

The marital status coefficient was both positive and statistically significant in influencing household food insecurity. The result’s implication is explained by the fact that spending on nutritious food is inversely correlated to the family head’s marital status. This finding conflicts with that of Maziya et al. [46], who discovered that the head of a household’s marital status was inversely correlated with food insecurity and that there is a lower likelihood of food insecurity if married people head a household [46]. However, the findings of this study are consistent with those of Ndlovu et al. [39], which found that unmarried household heads are more likely to have food security than married household heads. This could be explained by the fact that married households typically have larger households because they have more children and, therefore, more people to feed. As a result, there is much less food accessible to household members.

The regression analysis results also showed a significant and positive correlation between the number of dependents and household food insecurity. This suggests that food insecurity increases with household size. Large households have six or more dependents, while small households have any number of dependents under six. In this study, each family had an average of eight members; most households were large and food insecure. The fact that most of the household members were not employed may have contributed to these findings. This outcome is consistent with the research of Cheteni et al. [36], who discovered that having many economically inactive family members increases the likelihood of having unhealthy diets and nutrition. A large household size exerts pressure on the availability and accessibility of food because there are more food and non-food expenses.

## 5 Conclusions and Recommendations

Trees and green spaces play an important role in sustaining livelihoods and promoting food security in South African communities; however, their role has not been widely accepted. This study’s findings indicate that trees and green spaces are contributing considerably to the food and nutrition security of the local households studied. Thus, there is need for deliberate investments and public awareness campaigns recognising the significance of all trees and green spaces to food and nutrition security. That would most likely reduce the pressure put on cultivated cash crops to ensure food security.

There is potential for food security solutions to be formed around cultivated and uncultivated green spaces to promote sustainable access to food and nutritious diets in low-income households. Improvement in education around the benefits of trees and green can also assist in alleviating food insecurity.

Given the foods found in trees and green spaces around the communities, more research is required to develop knowledge in this area and an in-depth evaluation of household diets. This can help understand which nutritious foods can be cultivated and harvested from trees and green spaces, given that they have lower impacts on the ecosystem. Policy interventions should include promotion of incorporation of foods from both cultivated and uncultivated (wild) trees and green spaces in the diets of the local households. That could be done through skills development, raising awareness on the importance of consuming balanced diets, and promoting the sustainable use of natural resources. Further research could also help identify the possibility of cooperation between extension workers, nutritionists, policymakers, and other stakeholders to develop holistic, sustainable solutions to the food and nutrition insecurity problem. This can create an enabling environment for better policies and interventions, and improve households’ access to nutritious, high-quality, sustainable foods.

## 6 Limitations

The study only collected data that answered the study’s objectives; for future research, it is important to broaden the scope and look at how technologies improve food access from trees and green spaces. Furthermore, the study focused on one province, Kwa-Zulu-Natal. To draw lessons from one province to another, the research should be expanded to cover other areas in KwaZulu-Natal and all provinces in South Africa. The current study was conducted to establish the contribution of trees and green spaces on household food security. Further studies could be done to assess the effect of trees and green spaces on household nutrition security and income. Future research could also focus on food preferences, coping strategies, and assisting food-insecure households in accessing nutritious foods from trees and green spaces.

## Figures and Tables

**Table 1:** The HFIAS survey questions.

Did Your Household Face the Following Problems in the Past Four Weeks	Yes		Rarely		Sometimes		Often	
	Freq.	%	Freq.	%	Freq	%	Freq.	%
Worry that your household would not have enough food?	175	62.5	87	31.1	72	25.7	16	5.7
Unable to eat the kinds of foods you preferred because of a lack of resources?	183	65.4	86	30.7	69	24.6	28	10
Eat a limited variety of foods due to a lack of resources?	170	60.7	81	28.9	73	26.1	18	6.4
Eat some foods that you did not want to eat because of a lack of resources to obtain other types of food?	170	60.7	81	28.9	73	26.1	18	6.4
Eat a smaller meal than you felt you needed because there was not enough food?	162	57.9	74	26.4	78	27.9	12	4.3
Eat fewer meals in a day because there was not enough food?	149	53.2	68	24.3	66	23.6	15	5.4
Was there ever no food to eat of any kind in your household because of a lack of resources to get food?	127	45.4	42	15	68	24.3	17	6.1
Go to sleep hungry because there was not enough food?	46	16.4	33	11.8	6	2.1	5	1.8
Go a whole day and night without eating anything because there was not enough food?	18	6.4	7	2.5	9	3.2	2	0.7

Source: Own analysis (Multiple responses were allowed).

**Table 2:** Socio-demographic profiles.

	Osindisweni	Maphephetheni
**Sex of the respondents**		
**Male**	18.4%	26.5%
**Female**	81.6%	73.5%
**Household composition**		
Nuclear	98.7%	94.1%
Polygamous	1.3%	5.9%
**Level of education**		
Primary	14.5%	20.6%
Secondary	63.2%	56.6%
Tertiary	13.2%	13.2%
No education	9.2%	9.3%
**Marital status**		
Married	65.8%	57.4%
Single	18.4%	23%
Separated	6.5%	7.4%
Widowed	9.2%	12.3%
**Source of regular meals**		
Trees and green spaces	12%	28.8%
Bought from markets	64%	51.7%
From friends and relatives	24%	19.5%
**Farming and gardening**		
Communal gardens	2%	2.9%
Backyard gardens	90.7%	93%
**Livestock**		
Cattle	17.3%	12.2%
Goats	34.7%	22.4%
Sheep	1.3%	1%
Poultry	40%	35.1%
**Income**	30%	23%
Friends and family	72%	41%
Social and government grants	-	2%
Crafts, medicine, and fuel-wood sales	-	2%
Livestock sales	-	1%
Crop, fruit, and vegetable sales	16%	16%
Pension	40%	46%
Part-time employment	15%	17%
Full-time employment		

Socio-demographic profiles of the respondents. Source: Own analysis (multiple responses were allowed).

**Table 3:** The determinants of the HFIAS using the Instrumental Variable Poisson model.

HFIAS	Coef.	St.Err.	dy/dx	Sth.	*p*-Value
Cultivated green spaces	–0.097	0.030	–0.691	0.215	0.001 **
Non-cultivated green spaces	0.140	0.030	0.992	0.211	0.000 ***
Local trees	0.059	0.014	0.422	0.101	0.000 ***
Wealth Index: Non-Agricultural Related Assets and Agricultural Related Assets	–0.146	0.022	–1.041	0.159	0.000 ***
Gender of household head	–0.186	0.054	–1.324	0.384	0.001 ***
Age of household head	0.006	0.002	0.040	0.011	0.000 ***
Marital status of household head	0.107	0.016	0.758	0.112	0.000 ***
Household size	0.012	0.009	0.088	0.063	0.162
Education level of the head of household	–0.072	0.027	–0.512	0.190	0.007 ***
Number of dependents	0.021	0.010	0.152	0.071	0.031 **
Monthly Income	0.000	0.000	–0.001	0.000	0.000 ***
Grants	–0.315	0.116	–2.239	0.825	0.007 ***
Access to training, agricultural assistance, extension, and advisory services	– 0.057	0.101	– 0.404	0.721	0.575
Constant	2.164	0.135	***		
Mean dependent var	7.107				
Pseudo r-squared	0.114				
Chi-square	314.296				
Akaike crit. (AIC)	2476.966				
Bayesian crit. (BIC)	2527.853				
Prob > chi2	0.000				

Results of the Instrumental Variable Poisson model (Source: Own analysis).

*** *p* < 0.01

** *p* < 0.05.

## Data Availability

The data presented in this study are available upon request from corresponding authors.
